# The first complete chloroplast genome sequence of *Vicia ramuliflora* (Fabaceae)

**DOI:** 10.1080/23802359.2019.1705196

**Published:** 2020-01-07

**Authors:** Cheng Xin, Qian Yang

**Affiliations:** Key Laboratory of Resource Biology and Biotechnology in Western China (Ministry of Education), College of Life Sciences, Northwest University, Xi’an, China

**Keywords:** *Vicia ramuliflora*, chloroplast genome, phylogenetic analysis

## Abstract

*Vicia ramuliflora* belongs to the Fabaceae. It is a perennial herb, with high economic value. The cpDNA of *V. ramuliflora* was 124,682 bp long with IR loss. It contains 109 genes, including 76 protein-coding genes, 29 tRNA genes, and 4 rRNA genes. The overall GC content is 35.1%. The phylogenetic tree indicates that *Vicia* species formed a monophyletic lineage with high bootstrap value. This study has provided new genome information for the phylogenetic analysis of Fabaceae.

Fabaceae is an eudicotyledonous plant including over 18,000 species. It is the third largest family of seed plants and widely distributed throughout the world. *Vicia ramuliflora* belongs to the family Fabaceae. It is a perennial herb, with high economic value. Its nutrient content is as high as 26.9%. Its medicinal value is to tonify the kidney and regulate menstruation. Regarding the classification system of the *V. ramuliflora*. Having been controversial for more than 200 years and changed a lot. Therefore, in this study, we determined the complete chloroplast genome sequences of *V. ramuliflora* to provide new genome information for the phylogenetic analysis of Fabaceae.

Total genomic DNA was isolated from a single individual of *V. ramuliflora*. This sample was collected from Benxi (Liaoning, China; 41.11°N, 124.32°E), and the voucher specimen (2018XIN93) was deposited in the Evolutionary Botany Laboratory (EBL), Northwest University. Genomic DNA was isolated from the silica-dried leaves of a single individual with the improved CTAB method (Doyle 1987) and sequenced by using the Illumina HiSeq 2500 platform. Raw reads were trimmed by NGSQC Toolkit_v.2.3.3 (Patel and Jain 2012) and the clean reads were assembled by MITObim v1.8 (Hahn et al. 2013). The complete chloroplast genome was annotated by Geneious R8.0.2 (Biomatters Ltd., Auckland, New Zealand) with *Vicia sepium* (NC039595) as the reference. The annotated genome has been deposited into GenBank with the accession number of MN758738.

The cpDNA of *V. ramuliflora* was 124,682 bp long with IR loss. It contains 109 genes, including 76 protein-coding genes, 29 tRNA genes, and 4 rRNA genes. The overall GC content is 35.1%. Phylogenetic relationships were presented using 21 published species. Their whole chloroplast genome sequences were aligned with the program MAFFT 7.308 (Katoh and Standley 2016) and adjusted manually. Maximum likelihood (ML) analyses were implemented in RAxML version 7.2.6 with 1000 bootstrap replicates (Stamatakis 2006). The phylogenetic tree indicates that *Vicia* species formed a monophyletic lineage with high bootstrap value (Figure 1).

**Figure 1.**
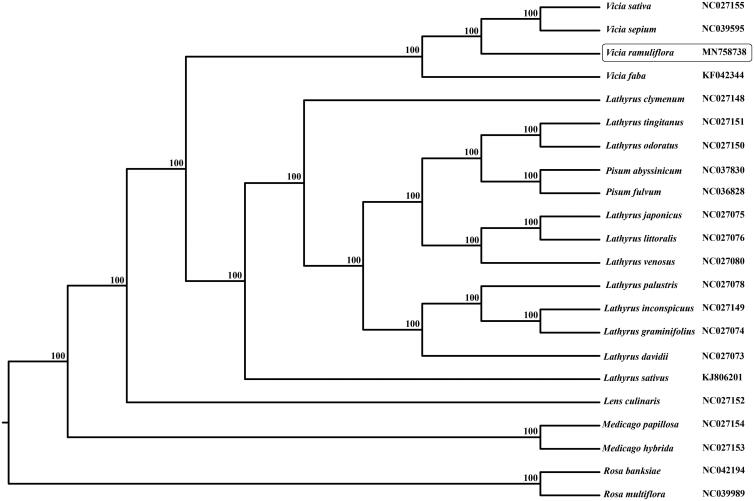
The phylogenetic tree based on complete chloroplast genome sequences.
